# Phenotyping of P105-Negative B Cell Subsets in Patients with Systemic Lupus Erythematosus

**DOI:** 10.1155/2012/198206

**Published:** 2011-09-26

**Authors:** Syuichi Koarada, Yoshifumi Tada, Rie Suematsu, Sachiko Soejima, Hisako Inoue, Akihide Ohta, Kohei Nagasawa

**Affiliations:** ^1^Division of Rheumatology, Faculty of Medicine, Saga University, 5-1-1 Nabeshima, Saga 849-8501, Japan; ^2^Division of Clinical Nursing, Faculty of Medicine, Saga University, 5-1-1 Nabeshima, Saga 849-8501, Japan

## Abstract

This study aimed to investigate phenotype of RP105(−) B cell subsets in patients with systemic lupus erythematosus (SLE). Flow cytometry was used for phenotyping RP105-negaive B cell subsets. Based on CD19, RP105, and CD138 expression, RP105(−) B cells consist of at least 5 subsets of late B cells, including CD19(+)RP105(int), CD19(+) RP105(−), CD19(low) RP105(−) CD138(−), CD19(low) RP105(−)CD138(int), and CD19(low) RP105(−) CD138(++) B cells. Especially, CD19(+)RP105(int) and CD19(low) RP105(−)CD138(int) B cells are significantly larger than other RP105(−) B cell subsets in SLE. By comparison of RP105(−) B cell subsets between patients with SLE and normal subjects, these subsets were detectable even in normal subjects, but the percentages of RP105(−) B cell subsets were significantly larger in SLE. The phenotypic analysis of RP105(−) B cell subsets suggests dysregulation of later B cell subsets in SLE and may provide new insights into understanding regulation of B cells in human SLE.

## 1. Introduction

Systemic lupus erythematosus (SLE) is a typical systemic autoimmune disease characterized by production of various autoantibodies including anti-double-strand (ds) DNA antibodies from B cells [1–4]. Although the pathogenesis of SLE is not fully clarified, autoantibody-producing B cells play a pivotal role in developing autoimmunity in SLE [3, 5]. Therefore, understanding of human B cell biology in autoimmune diseases is an essential issue.

RP105 (CD180) is one of the homologues of Toll-like receptors (TLRs). RP105 expresses on mature B cells, macrophages, and dendritic cells (DCs) [6]. It has been reported that RP105 is associated with activation of B cells in mice and humans [7, 8]. RP105 also facilitates macrophage activation by mycobacterium tuberculosis lipoproteins through TLR2 [9]. However, we and other investigators have reported that RP105 negatively regulates the signal of TLR4 in DCs [10, 11]. Although the function of RP105 is still controversial and undefined, RP105 may affect activation and function of B cells in immune systems.

We have previously reported that enlarged population of RP105-lacking [RP105(−)] B cells in peripheral blood (PB) is an outstanding feature in patients with active SLE [12, 13]. Although RP105(−) B cells may be assigned to be subsets of activated late B cells with producing immunoglobulins (Igs) and anti-dsDNA antibodies [14], precise phenotype has not been examined yet. 

Late B cells, including plasmablasts and plasma cells, play critical roles in humoral immune response and autoimmune diseases [15]. Comparison of the B cell subsets in healthy subjects with SLE patients could lead to relevant observations. The phenotypic analysis of subsets of RP105(−) B cells is helpful to understand the dysregulation of late B cells in SLE. 

## 2. Materials and Methods

### 2.1. Patients and Agents

Patients with active SLE (*n* = 15) (14 women and 1 man, mean ± SD age: 41.2 ± 10.5 years) were enrolled in this study, who fulfilled at least 4 of the 11 classification criteria for SLE as defined by the American College of Rheumatology [16] and as updated in 1997 [17]. None of the active SLE patients was receiving immuno-suppressive drugs at the time of examination. Age-matched 7 healthy volunteers joined as controls (6 women and 1 man, 38.2 ± 9.1 years). Written informed consent was obtained from all subjects prior to sample acquisition. The study protocol was approved by the Ethics Committees of Saga University, and the subjects' written consent was obtained according to the Declaration of Helsinki at the General Assembly in October 2008.

The following monoclonal antibodies (mAbs) were used in our studies fluorescein isothiocyanate-(FITC-) conjugated, phycoerythrin- (PE-) conjugated, or allophycocyanin- (APC-) conjugated antihuman CD19, FITC-conjugated or PE-conjugated antihuman RP105, FITC- or PE-conjugated anti-CD19, anti-CD20, anti-CD22, anti-CD24, anti-CD27, anti-CD28, anti-CD30, anti-CD31, anti-CD38, anti-CD40, anti-CD62L, anti-CD70, anti-CD72, anti-CD77, anti-CD79b, anti-CD80, anti-CD86, anti-CD95, anti-CD97, anti-CD126, anti-CD138, anti-CD147, anti-CD164, anti-CD200, anti-CD209, anti-CD267, anti-CD275, anti-CD279, anti-CCR7, anti-CXCR5 (CD185), anti-HLA-DR, anti-IgG, anti-IgM, anti-IgD, anto-TLR5, anti-TLR6, PE-conjugated anti-CD10, anti-CD21, anti-CD23, anti-CD25, anti-CD27, anti-CD28, anti-CD45RO, anti-CD69, anti-CD77, anti-CD122, anti-CD125, anti-CD132, anti-CD150, anti-CD152, anti-CD184 (CXCR4), anti-CCR2, anti-CCR10, anti-CX40, and anti-TLR2 were purchased from BD Bioscience (San Jose, CA, USA). The mAbs to human BCMA (B cell maturation antigen) (Vicky-1, rat IgG1), BAFF-R (B cell activating factor receptor) (11C1, mouse IgG1), and TACI (transmembrane activator and calcium modulator ligand interactor; CD267) (1A1, rat IgG2a) were obtained from ALEXIS Biochemical (Piscataway, NJ, USA). FITC- or PE-conjugated isotype-matched control mAbs were purchased from BD Bioscience. PerCP- (Peridinin chlorophyll protein-) conjugated CD138 was also obtained from BD Bioscience.

### 2.2. Flow Cytometric Analysis

Heparinized peripheral venous blood was obtained from patients with SLE. PB mononuclear cells (PBMCs) were separated immediately by centrifugation over Ficoll-Hypaque (Pharmacia Biotech, Uppsala, Sweden). PBMCs were washed twice and resuspended at 1 × 10^6^ cells/mL in staining buffer.

Direct immunofluorescence was carried out with PE- or FITC-conjugated antibodies against surface antigens and stained with FITC- or PE-conjugated anti-RP105, PerCP-conjugated anti-CD138, and APC-conjugated anti-CD19 mAbs. Irrelevant isotype-matched control antibodies were used to determine background fluorescence. These samples were analyzed with the saved setting of gate. More than 500 000 viable, antibody-labeled cells were identified according to their forward and side scattering, electronically gated, and analyzed on a FACScalibur flow cytometer (Becton Dickinson). Results were expressed as percent of positive cells or mean fluorescence intensity (MFI) using WINMDI software (http://facs.scripps.edu/software.html). The percentages of subsets of RP105(−) B cells (RP105(−) CD19(+) subset cells/CD19(+) cells%) were calculated.

### 2.3. Statistical Analysis

Statistical analysis was performed with the Mann-Whitney *U* test, the Wilcoxon signed rank test, or Student's *t*-test using SPSS software (SPSS Japan Inc., Tokyo, Japan); statistical significance was considered with a *P* < 0.05.

## 3. Results

### 3.1. RP105-Negative B Cells Consist of 5 Subsets of B Cells

**Figure 1:**
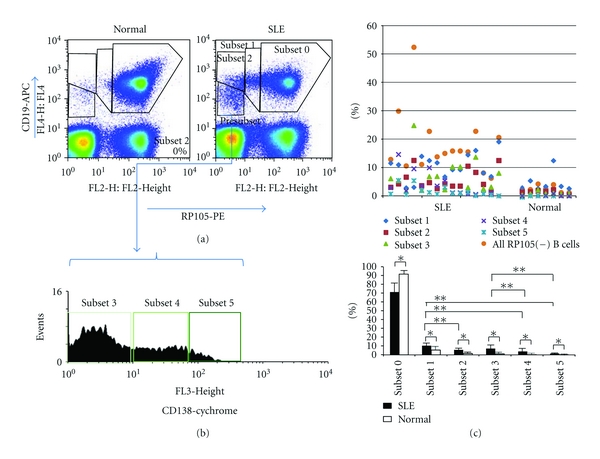
Subsets of RP105(−) B cells. (a) Representative flow cytometric profiles of RP105 expression on CD19(+) B cells from an active SLE patient and a normal subject. The population was subdivide (1) subset 1; CD19(+)RP105(int), (2)subset 2; CD19(+)RP105(−), and (3) presubset; CD19(low)RP105(−). (b) CD138 levels after gating CD19(low)RP105(−) presubset. The presubset cells were further subdivided into 3 subpopulations, subset 3; CD138(−), subset 4; CD138(int) and subset 5; CD138(++). (c) The percentages of subsets in PB of each patient and normal subject. *P < 0.05,  **P < 0.05.

In SLE patients, there is a large population of RP105(−) B cells [12].  Figure 1(a) shows a representative FACS profile of CD19 and RP105 expression on PBMCs from a patient with SLE and a normal subject. As reported in the previous studies [12, 13], a significant population of CD19(+) B cells from SLE patients lacked RP105. In this study, in conjunction with RP105, we used the B cell marker CD19 and plasma cell marker CD138 in an attempt to subdivide RP105(−) B cells further.

We identified 4 populations of B cells in the panel of CD19 and RP105. The populations were CD19(+)RP105(+) B cells (subset 0) and three RP105(−) B cell subpopulations, CD19(+)RP105(int; intermediate) (subset 1), CD19(+)RP105(−) (subset 2), and CD19(low)RP105(−) B cells (presubset). The population of CD19(low)RP105(−) B cells (presubset) was further subdivided into 3 subpopulations, CD19(low)RP105(−)CD138(−) (subset 3), CD19(low)RP105(−)CD138(int) (subset 4), and CD19(low)RP105(−)CD138(++) (subset 5) B cells according to CD138 expression levels after gating presubset (Figure 1(b)). Collectively, we identified at least 5 subsets of RP105(−) or (low) B cells.

The percentages of subsets in PB of each patient and normal subject were shown in Figure 1(c). Among these subsets, the populations of subset 1 (10.4 ± 4.4%) and 3 (7.1 ± 5.8%) B cells were larger than other subsets (subset 2; 5.0 ± 3.5%, subset 4; 3.8 ± 4.2%, subset 5; 1.6 ± 1.7%). All RP105-negative B cell subsets of SLE patients were significantly increased compared with normal subjects.

### 3.2. Phenotype of RP105-Negative B Cell Subsets in SLE

**Figure 2:**
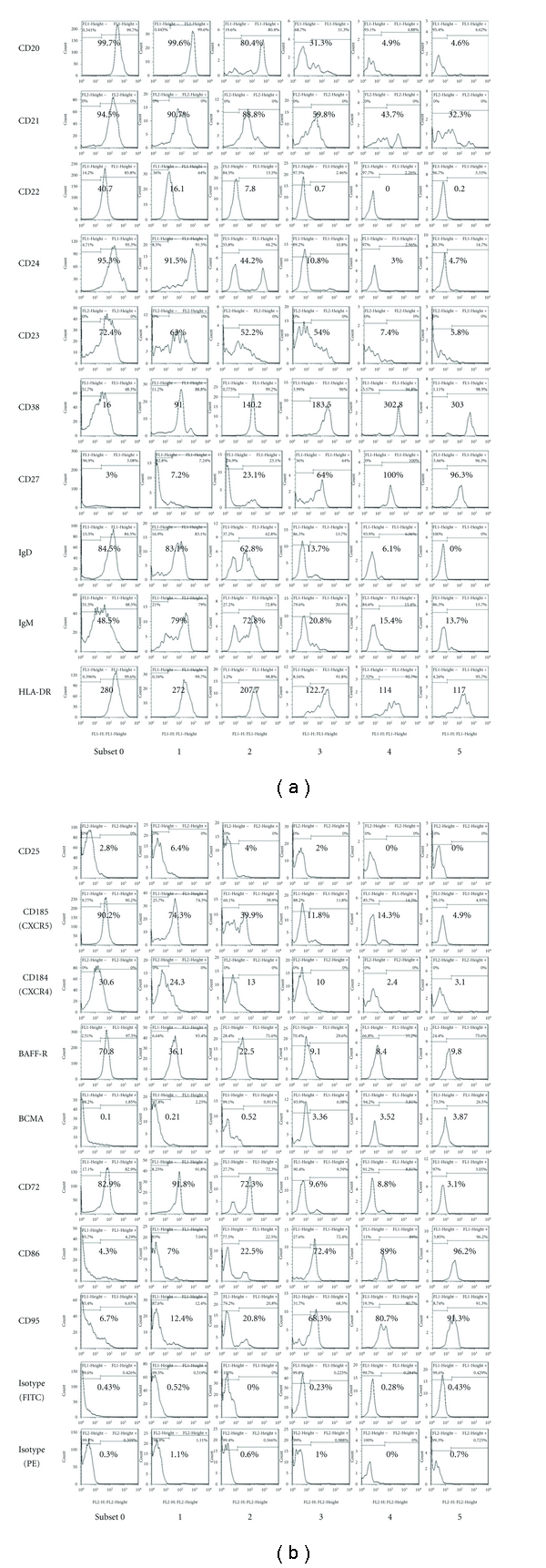
Flow cytometric analysis of various antigens on and in B cell subsets from a patient with SLE. Positive cell ratio or MFI (mean fluorescence intensity) was shown in flow cytometric profiles.

**Figure 3:**
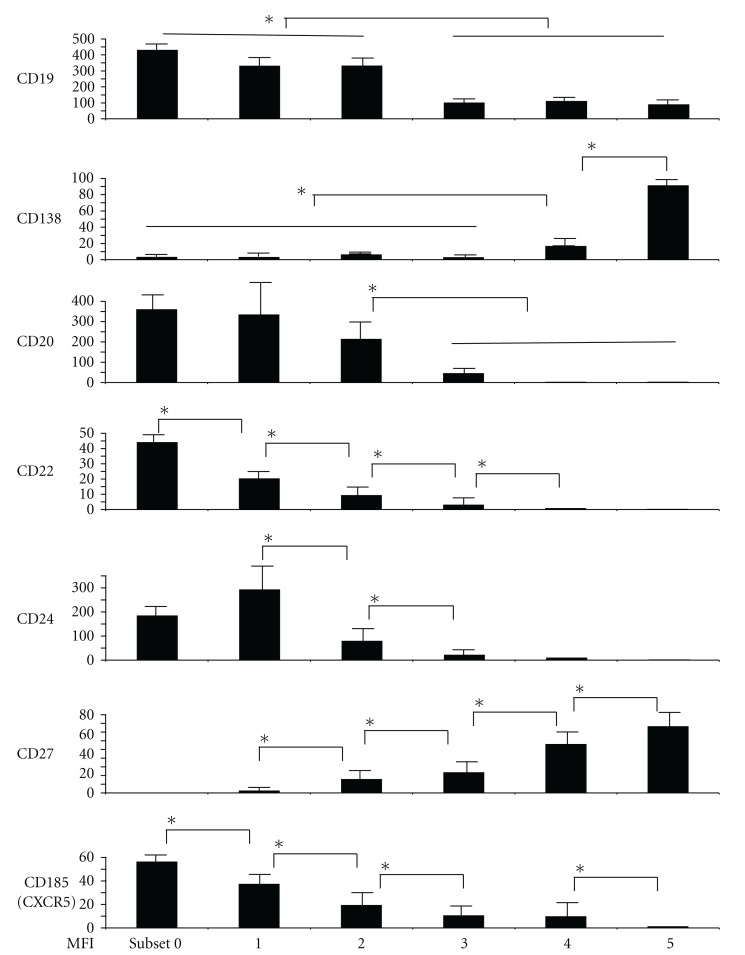
Expressions of important B cell markers (MFI) on RP105(−) B cells (subset 1–5) and RP105(+) B cells (subset 0). The levels of CD19, CD20, and CD24 were significantly lower in subset 3, 4, and 5 B cells compared to subset 0, 1, and 2 B cells (*P* < 0.05). CD27 and CXCR5 expression was significantly different: (*P* < 0.05 subset 0 versus 1, subset 1 versus 2, subset 2 versus 3, subset 3 versus 4, and subset 4 versus 5).

In order to characterize these subsets, we analyzed various antigens on B cells (Figures 2 and 3). Subset 0, 1, and 2 B cells were positive for CD20, but in subset 3, 4, and 5 B cells, CD20 expression decreased. Although subset 0, 1, and 2 B cells expressed CD21, CD22, and CD24, subset 3, 4, and 5 B cells lost CD22 completely, and expression levels of CD21 and CD24 were lower compared to subset 0, 1, and 2 B cells. CD23 expression was low only in subset 4 and 5 B cells.

On the other hand, CD38 and CD27 expressions were the lowest on subset 0 B cells. The levels of CD38 and CD27 gradually increased from subset 1 to subset 4 and 5 B cells. While subset 0, 1, and 2 B cells expressed surface IgD and IgM, subset 3, 4, and 5 B cells had lower levels of surface Igs. All the subsets of RP105(−) B cells expressed HLA-DR. However, the levels of HLA-DR gradually decreased. Low expression of CD25 was detectable in subset 0, 1, 2, and 3 B cells.

CXCR5 (CD185), a homing receptor, presented in subset 0, 1, and 2 B cells, but it disappeared in the subset 3, 4, and 5 B cells. The expression of CXCR4 (CD184) was the highest in subset 0 B cells but was lost in subset 4 and 5 B cells.

More interestingly, of BAFF receptors, BAFF-R expression was higher in subset 0, 1, and 2 B cells, but BCMA expression was conversely higher in subset 3, 4, and 5 B cells.

From subset 0 to subset 2 B cells, CD72, CD79b, and CD200 expressions were found, but those were lower on subset 3, 4, and 5 B cells. CD86, CD95, CD97, and CD126 were positive on subset 3, 4, and 5 cells. CD1a, CD1b, CD10, CD40, CD77, and CD80 were low or negative in all subsets. CD31, CD49d, and CD45RA were constantly positive on all subsets (data not shown).

We investigated and summarized the phenotype of RP105(−) B cell subsets in patients with active SLE. RP105(−) B cells consist of at least 5 subsets of late B cells, including CD19(+)RP105(int), CD19(+)RP105(−),  CD19(low)RP105(−)CD138(−), CD19(low)RP105(−)CD138(int),  and  CD19(low)RP105-(−)CD138(++) B cells. The phenotypic analysis of RP105(−) B cells suggests mature phenotype of these RP105(−) B cells, CD20(low or lost) CD22(low or lost) CD27(high) CXCR5(low or lost).

### 3.3. Phenotype of RP105-Negative B Cell Subsets in Normal Subjects

We analyzed whether our finding of subsets of RP105(−) B cells is valid in healthy subjects. Although circulating RP105(−) B cells are very rare in healthy subjects, subsets of RP105(−) B cells were identified. Therefore, comparison of the identified B cell subsets in healthy subjects with SLE patients could lead to relevant observations.

**Figure 4:**
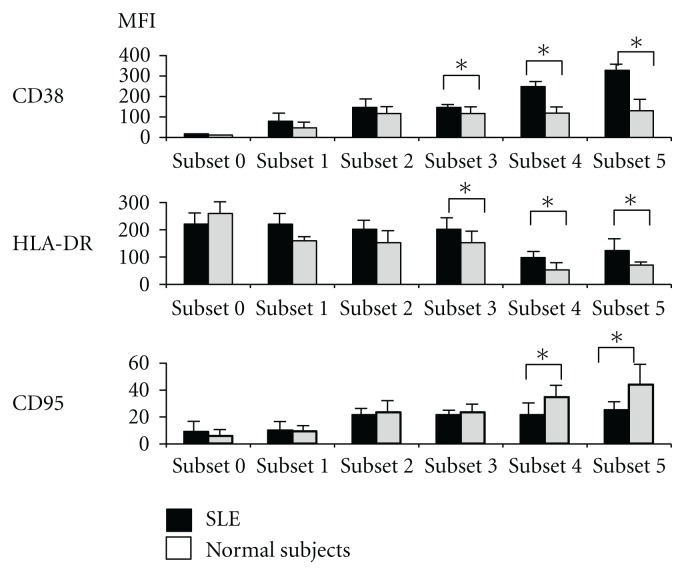
Comparison of surface expression of various antigens with significant difference of mean of MFI on RP105(−) B cell subsets between SLE patients and normal subjects.

Virtually, patterns of phenotype of RP105(−) B cells from normal subjects seemed similar to those from SLE patients. However, the expression levels of several antigens were significantly different between SLE patients and normal subjects (Figure 4). In subset 3, 4, and 5, levels of CD38 and HLA-DR of SLE patients were significantly higher than those of normal subjects (*P* < 0.05). On the other hand, in subset 4 and 5, levelsofCD95 were lower in SLE patients compared with normal subjects (*P* < 0.05).

## 4. Discussion

RP105 (CD180) is mainly expressed on mature B cells and regulates B cell function in humans and mice [6]. Enlarged population of RP105(−) B cells was remarkable in active patients with SLE [12]. In the past studies we suggested that RP105(−) B cells are activated and well-differentiated B cells. However, precise phenotype of RP105(−) B cells has not been elucidated. We described here phenotype of human late B cells more precisely using markers of CD19, RP105, and CD138.

We identified 5 subpopulations of RP105(−) B cells with different phenotype that are categorized as follows: subset (1) CD19(+)RP105(int), (2) CD19(+)RP105(−), (3) CD19(low)RP105(−)CD138(−), (4) CD19(low)RP105(−)CD138(int), and (5) CD19(low)RP105(−)CD138(++) B cells. Each subset of RP105(−) B cells showed different phenotype of B cells.

IgD and CD38 were classically used to subdivide PB B cells into four quadrants, IgD(+)CD38(−) naïve B cells (Bm1 and Bm2), IgD(+)CD38(+) Pre-GC B cells (Bm2'a and Bm2'b), IgD(−)CD38(+) GC B cells (Bm3 and Bm4), and IgD(−)CD38(−) memory B cells (Bm5) [18]. According to this classification system, subset 3, 4, and 5 of RP105(−) B cells, expressing CD38 and lacking surface IgD, may be tentatively considered to belong to GC B cells. Although GC B cells are CD10(+), CD20(++), and CD27(−), the RP105(−) B cells lost CD10 and CD20, but express CD27. Therefore, several subsets of RP105(−) B cells do not correspond with classically categorized GC type B cells. 

CD27 expression is commonly used as an exclusive marker for human memory B cells [18]. Although memory B cells express CD27 and lack IgD and CD38, 5 subsets of RP105(−) B cells clearly expressed CD38. Accordingly, RP105(−) B cells are not memory type B cells. Several previous reports have shown that CD27-bright plasma cells increasehighlyin PBMC from patients with active SLE and that the frequency is useful in evaluating disease activity with a significant correlation with SLEDAI and autoantibodies [19, 20]. We presented CD27 expression of RP105-negative B cell subsets in Figures 2 and 3. CD27 expression was gradually increased from subset 0 to subset 5. It has been reported that CD27 expression is upregulated during B cells differentiation into plasma cells [21]. Therefore, higher expression of CD27 on B cells is also a marker of differentiation towards plasma cells [21]. Based on CD20, CD27, and CD38 expressions, RP105(−) B cells correspond neither to germinal center B cells nor memory B cells.

It is reported that in vitro human activated B cells and plasmablasts/plasma cells express CD19, whose levels, however, are lower on plasmablasts and plasma cells compared to activated B cells [22]. Actually, in this study, CD19 expression was lower in subset 3, 4, and 5 B cells.

Regarding homing receptors, differentiated plasma cells are characterized by a disappearance of CXCR5, a progressive reduction in CXCR4 [22]. From subset 0 to subset 5 B cells, CXCR5 and CXCR4 levels became decreased. The expression levels of these molecules also suggested that RP105(−) B cell subsets may belong to the cells during the process towards plasma cell formation.

RP105(−) B cells have abundant intracellular Igs [12] but lost surface Igs. Therefore, subset 3, 4, and 5 B cells may be assigned as to be plasmablasts or plasma cells, preparing to secret Igs. However, due to lacking CD138, subset 3 B cells may be assigned as plasmablasts not as plasma cells. On the other hand, subset 4 B cells may be assigned as preplasma cells due to intermediate CD138 expression and subset 5 B cells as PB plasma cells with bright CD138.

Collectively, each subset represents the step of B cell differentiation towards plasma cells. However, to confirm each steps comparison between PB and bone marrow plasma cells is required. Further analysis of in vitro culture of B cells that develop and differentiate into plasma cells with morphological change, functional studies, and expression of transcription factors is also important. In our results, we present the existence of increased various late B cell subsets in SLE patients.

We performed phenotype analysis in healthy subjects. The results were shown in Figures 1 and 4. Although circulating RP105(−) B cells are very rare, we found the subsets of RP105(−) B cells even in healthy subjects. The comparison of the identified B cell subsets in healthy subjects with SLE patients showed that, virtually, phenotype of RP105(−) B cells from normal subjects seemed similar to those from SLE patients. Interestingly, the expression levels of several antigens were different between SLE and normal subjects. However, the importance of the expression levels of antigens for pathophysiology in SLE is not unclear in this study. Therefore, further analysis of the antigens of RP105(−) subsets found in SLE patients with various activity and other systemic rheumatic diseases should be required.

Recently, it has been reported that increased circulating CD138(int) B cells, producing autoantibodies, are related to autoimmunity in MRL/lpr lupus mice [15]. RP105(−) B cells showed similar phenotype with CD138(int) B cells: (1) intermediate expression of CD138, (2) larger cell size and increased granularity [12], (3) cytoplasmic Ig and IgM expression. Thus, CD19(low)RP105(−)CD138(int) B cells may be the human counterparts of CD138(int) B cells in mice. These results suggest possible similar mechanisms in dysregulation of B cells in human and murine autoimmune diseases.

Further studies will be required to determine mechanisms of appearing, differentiation, and proliferation of RP105(−) B cell in human SLE. It is possible that inappropriate enlarged population of various subsets of RP105(−) B cells in PB is greatly related to pathophysiology in SLE.

##  Conflict of Interests

No conflict of interests has been declared by the authors.

## References

[1] Hahn BH (1998). Antibodies to DNA. *New England Journal of Medicine*.

[2] von Muhlen CA, Tan EM (1995). Autoantibodies in the diagnosis of systemic rheumatic diseases. *Seminars in Arthritis and Rheumatism*.

[3] Lipsky PE (2001). Systemic lupus erythematosus: an autoimmune disease of B cell hyperactivity. *Nature Immunology*.

[4] Pisetsky DS (2000). Anti-DNA and autoantibodies. *Current Opinion in Rheumatology*.

[5] Mitchison NA, Wedderburn LR (2000). B cells in autoimmunity. *Proceedings of the National Academy of Sciences of the United States of America*.

[6] Miyake K, Yamashita Y, Hitoshi Y, Takatsu K, Kimoto M (1994). Murine B cell proliferation and protection from apoptosis with an antibody against a 105-kD molecule: unresponsiveness of X-linked immunodeficient B cells. *Journal of Experimental Medicine*.

[7] Nagai Y, Shimazu R, Ogata H (2002). Requirement for MD-1 in cell surface expression of RP105/CD180 and B-cell responsiveness to lipopolysaccharide. *Blood*.

[8] Ogata H, Su I, Miyake K (2000). The toll-like receptor protein RP105 regulates lipopolysaccharide signaling in B cells. *Journal of Experimental Medicine*.

[9] Blumenthal A, Kobayashi T, Pierini LM (2009). RP105 Facilitates Macrophage Activation by Mycobacterium tuberculosis Lipoproteins. *Cell Host and Microbe*.

[10] Tada Y, Koarada S, Morito F (2008). Toll-like receptor homolog RP105 modulates the antigen-presenting cell function and regulates the development of collagen-induced arthritis. *Arthritis Research and Therapy*.

[11] Divanovic S, Trompette A, Atabani SF (2005). Negative regulation of Toll-like receptor 4 signaling by the Toll-like receptor homolog RP105. *Nature Immunology*.

[12] Koarada S, Tada Y, Ushiyama O (1999). B cells lacking RP105, a novel B cell antigen, in systemic lupus erythematosus. *Arthritis and Rheumatism*.

[13] Koarada S, Ide M, Haruta Y (2005). Two cases of antinuclear antibody negative lupus showing increased proportion of B cells lacking RP105. *Journal of Rheumatology*.

[14] Kikuchi Y, Koarada S, Tada Y (2002). RP105-lacking B cells from lupus patients are responsible for the production of immunoglobulins and autoantibodies. *Arthritis and Rheumatism*.

[15] Culton DA, O'Conner BP, Conway KL (2006). Early preplasma cells define a tolerance checkpoint for autoreactive B cells. *Journal of Immunology*.

[16] Tan EM, Cohen AS, Fries JF (1982). The 1982 revised criteria for the classification of systemic lupus erythrematosus. *Arthritis and Rheumatism*.

[17] Hochberg MC (1997). Updating the American College of Rheumatology revised criteria for the classification of systemic lupus erythematosus. *Arthritis and Rheumatism*.

[18] Agematsu K, Hokibara S, Nagumo H, Komiyama A (2000). CD27: a memory B-cell marker. *Immunology Today*.

[19] Jacobi AM, Odendahl M, Reiter K (2003). Correlation between circulating CD27^high^ plasma cells and disease activity in patients with systemic lupus erythematosus. *Arthritis and Rheumatism*.

[20] Dörner T, Lipsky PE (2004). Correlation of circulating CD27^high^ plasma cells and disease activity in systemic lupus erythematosus. *Lupus*.

[21] Jung J, Choe J, Li L (2000). Regulation of CD27 expression in the course of germinal center B cell differentiation: the pivotal role of IL-10. *European Journal of Immunology*.

[22] Jourdan M, Caraux A, de Vos J (2009). An in vitro model of differentiation of memory B cells into plasmablasts and plasma cells including detailed phenotypic and molecular characterization. *Blood*.

